# Supplementary material for: Potential Prognostic Markers for Relapsed/Refractory vs. Responsive Acute Myeloid Leukemia
Source: Cancers (Basel). 2022 Jun 1;14(11):2752. doi: 10.3390/cancers14112752 (PMC9179343; doi:10.3390/cancers14112752)
Supplement: Supplementary file 1 [file cancers-14-02752-s001.zip › cancers-1723799-supplementary.pdf]

Supplementary Table S1. Diagnosis and treatment strategies of AML patients. Response evaluation was based on the European Leukemia Network (ELN) 2017 recommendations.

Patient No.	Diagnosis	Molecular Genetics	Karyotype	ELN2017 risk group	Treatment and best response	Disease status
**Treatment responsive patients (n=15)**						
1	APL	FLT3-ITD, PML-RARA	t(15;17)	Favorable	PETHEMA/HOVON LPA 2005: AIDA induction + Consolidation + Maintenance – CRm.	CRm
2	APL	FLT3-ITD, PML-RARA	t(15;17)	Favorable	NA	CRm
3	APL	FLT3-ITD, PML-RARA	t(15;17)	Favorable	PETHEMA/HOVON LPA 2005: AIDA induction + Consolidation + Maintenance – CRm.	CRm
4	AML, not otherwise specified	NPM1, FLT3-ITD	Normal	Intermediate	7+3 induction - CRm. MUD alloSCT	CRm
5	AML, not otherwise specified	NPM1, FLT3-ITD, IDH2	Normal	Favorable	7+3 induction - CR MUD HLA10/10 alloSCT - CRm was confirmed.	CRm
6	AML, not otherwise specified	FLT3-ITD, IDH2, DNMT3A	Normal	Adverse	7+3 - RD FLAG-Ida - CR. MRD alloSCT Maintenance with Midostaurin, Sorafenib.	CRm
7	De Novo AML, NOS	IDH2	Normal	Intermediate	7+3 induction - CR 2 cycles of HDAraC MUD alloSCT.	CR
8	AML-MRC, secondary post MDS	IDH2	Normal	Intermediate	7+3 – RD FLAG-Ida - CR. MUD alloSCT.	CR
9	AML-MRC, secondary post MDS	None	Normal	Intermediate	7+3 induction - CR. MRD alloSCT	CR
10	AML-MRC, secondary post MDS	None	Normal	Intermediate	7+3 induction - CR 1 cycle of HDAraC + Daunorubicin Haploidentical alloSCT.	CR

11	De Novo AML, NOS.	IDH2	Normal	Intermediate	7+3 - RD,<br>FLAG-Ida - CR<br>MUD alloSCT.	CR
12	De Novo AML with recurent genetic abnormalities	None	t(8;21)(q22;q22), AML1/ETO	Favorable	7+3 – RD<br>HDAraC + Daunorubicin – CR.	CR
13	De Novo AML with recurent genetic abnormalities	NPM1, FLT3-ITD	Normal	Intermediate	7+3 induction – CR<br>1 cycle of HDAraC + Daunorubicin<br>MUD alloSCT.	CR
14	AML-MRC, secondary post MDS	SF3B1	Complex (del 5q, 19p, dup 3q)	Adverse	FLAG-Ida induction - CR	CR
15	De Novo AML with recurent genetic abnormalities	NPM1	Normal	Favorable	7+3 induction – CR<br>1 cycle of HDAraC + Daunorubicin<br>Autologous SCT.	CR
**Treatment refractory patients (n=16)**						
16	AML, not otherwise specified	FLT3-ITD, ASXL1	Normal	Adverse	Decitabine + Ibrutinib - RD.<br>Low Dose Cytarabine - PD	RD
17	Secondary AML, with myelodysplasia related changes	FLT3-ITD	Trisomy 14	Adverse	Decitabine + Ibrutinib - RD.<br>7+3 - RD	RD
18	AML, not otherwise specified	FLT3-ITD	Normal	Intermediate	7+3 induction - PR<br>HDAraC + Daunorubicin – response was not evaluated, death in aplasia.	PR
19	AML-MRC, secondary post MDS	NPM1, TET2	Normal	Favorable	Decitabine - RD<br>ACTIVE - CR<br>Palliation therapy LDAraC + Glasdegib for relapsed disease - PD	PD
20	AML-MRC, secondary post MDS	TP53, DNMT3A	Complex (del 5q, 7p, 13q, 16p, 17p, +8)	Adverse	7+3 - CR.<br>MUD alloSCT.<br>ACTIVE treatment for relapsed disease - CR.<br>Palliation with LDAraC + Glasdegib for second relapse – PD.	PD

21	AML-MRC	PTPN11, CUX1, SMC3, CBL, RUNX1;	del(11p), 11q23 MLL (KMT2A) rearrangement	Adverse	7+3 - RD, HDAraC + Daunorubicin - CR. MRD alloSCT. ACTIVE for relapsed disease - RD. Palliation with LDAraC + Glasdegib - PD	PD
22	AML-MRC, secondary post MDS	TET2	Normal	Intermediate	Decitabine - RD. ACTIVE – MLFS was achieved, death in aplasia.	MLFS
23	AML-MRC, secondary post MDS	None	del(5q)	Adverse	Decitabine - RD. ACTIVE - CRi	CRi
24	AML-MRC, secondary post MDS	None	del(3p), del(5q), del(9q), del(12q), del(17p)	Adverse	FLAG-Ida - RD ACTIVE - RD Decitabine + Venetoclax - RD LDAraC + Glasdegib - RD	RD
25	AML-MRC, secondary post MDS	ASXL1, IDH1	Normal	Adverse	7+3 - RD, HDAraC - RD ACTIVE - RD	RD
26	AML, secondary, therapy related	ASXL1, NRAS	Normal	Adverse	7+3 - RD, FLAG-Ida - RD ACTIVE - RD LDAraC + Glasdegib - CR. MUD alloSCT.	CR
27	AML-MRC, secondary post MDS	WT1, PTPN11	del(7q)	Adverse	Decitabine - RD. FLAG-Ida - CRi. MUD alloSCT LDAraC + Venetoclax for relapse - PD	PD
28	AML-MRC	None	del(7q)	Adverse	7+3 - RD LDAraC + Venetoclax - MLFS was achieved, death in aplasia	MLFS
29	AML-MRC	KIT, KRAS	-7, dup(3q)	Adverse	7+3 - RD, FLAG-Ida - RD Actinomycin D + Venetoclax - RD Decitabine + Venetoclax - RD Sorafenib - RD	RD
30	AML-MRC, secondary post MDS	TP53	Complex (48,XX,+1,+8,+11,-6,del(5q)	Adverse	7+3 - RD, HDAraC + Daunorubicin - RD. Salvage LDAraC + Venetoclax - CR MUD alloSCT	CR
31	AML-MRC, secondary post MDS	PTPN11, EZH2, RUNX1	dup(15q), LOH(q11)	Adverse	7+3 - RD FLAG-Ida - RD Actinomycin D + Venetoclax, 2 cycles - RD	RD

Abbreviations: ACTIVE – low dose cytarabine, actinomycin D, venetoclax; AlloSCT – allogeneic stem cell transplantation; AIDA induction – Idarubicin, ATRA; AML – acute myeloid leukemia; AML-MRC – acute myeloid

leukemia with myelodysplasia related changes; AML NOS – acute myeloid leukemia not otherwise specified; APL – acute promyelocytic leukemia; CR – complete remission; CRi – complete remission with incomplete hematological recovery; CRm – complete remission with negative minimal residual disease by multicolored flow cytometry; FLAG-Ida – Fludarabine, Cytarabine, Idarubicin, G-CSF; HDAraC – high dose Cytarabine; HLA – human leucocyte antigen; LDAraC – low dose cytarabine; MDS – myelodysplastic syndrome; MLFS – morphological leukemia free state; MRD – matched related donor; MUD – matched unrelated donor; NA – information not available; PD – progressive disease; PR – partial remission; RD – refractory disease; 7+3: cytarabine and daunorubicin.

Supplementary Table S2. Primers used for RT-qPCR analysis

Gene	Forward and reverse primers
ABCB1	F: GTCTGGACAAGCACTGAAA<br>R: AACAACGGTTCGGAAGTTT
APAF1	F: GGCTGTGGGAAGTCTGTATTAGC<br>R: ACTCTCATCCTGATCCAACCG
BAK1	F: TCATCGGGGACGACATCAAC<br>R: CAAACAGGCTGGTGGCAATC
BAX	F: TGCCTCAGGATGCGTCCACCAA<br>R: CCCCAGTTGAAGTTGCCGTCAG
BCL2	F: CGGAGGCTGGGATGCCTTTG<br>R: TTTGGGGCAGGCATGTTGAC
BCL2A1	F: TTACAGGCTGGCTCAGGACT<br>R: AGCACTCTGGACGTTTTGCT
BCL2L1	F: TGCATTGTTCCCATAGAGTTCCA<br>R: CCTGAATGACCACCTAGAGCCTT
BCL2L2	F: CTTGGTCTTGTTGTGAGTATGC<br>R: TGGAGCCGATGGTCTAGTC
BECN1	F: CTCCCGAGGTGAAGAGCATC<br>R: GCTGTTGGCACTTTCTGTGG
DAPK1	F: CAAGACAGGCACGGCAATAC<br>R: GGCTCCCATCAGACAGAGATAC
DNMT1	F: ACCGCTTCTACTTCCTCGAGGCCTA<br>R: GTTGCAGCTCTCTGTGAACACTGTGG
DNMT3A	F: CAGCGTCACACAGAAGCATATCC<br>R: GGTCCTCACTTTGCTGAACTTGG
EZH2	F: GTGGAGAGATTATTTCTCAAGATG<br>R: CCGACATACTTCAGGGCATCAGCC
GAPDH	F: AGTCCCTGCCACACTCAG<br>R: TACTTTATTGATGGTACATGACAAGG
GATAD2A	F: GACGGAGACATGAGGGTGAC<br>R: CGTTGTCGGAGAGCACAATCA
HDAC1	F: CAAGCTCCACATCAGTCCTTCC<br>R: TGCGGCAGCATTCTAAGGTT
HDAC2	F: AGTCAACGAGGCGGCAAAA<br>R: TGCGGATTCTATGAGGCTTCA
IDH1	F: TTGGCTGCTTGCATTAAAGGTT<br>R: GTTTGGCCTGAGCTAGTTTGA
IDH2	F: GCTGGAGAAGGTGTGCGTG<br>R: TGTTCAGGAAGTGCTCGTTCAG
KAT2A	F: CAGTTTCGGCAGAGGTCTCA<br>R: ATGAGTGGTTTCGTAGCGGG
KAT2B	F: CGAATCGCCGTGAAGAAAGC<br>R: CTTGCAGGCGGAGTACACT
KAT6A	F: TGTCAGTTTGGGGCATCTCC<br>R: TCTTATGCCGGGAGGAAGGA
KDM6B	F: TTCCTGTGTACCGCTTCGTG<br>R: AGCTGGTACTGATAGGCGGT

MCL1	F: GTGCCTTTGTGGCTAAACACT<br>R: AGTCCCGTTTTGTCCTTACGA
MYC	F: AATGAAAAGGCCCCCAAGGTAGTTATCC<br>R: GTCGTTTCCGCAACAAGTCCTCTTC
MTA1	F: AGCTACGAGCAGCACAACGGGGT<br>R: CACGCTTGGTTTCCGAGGAT
MTA2	F: TGTACCGGGTGGGAGATTAC<br>R: TGAGGCTACTAGAAATGTCCCTG
CDKN1A (p21)	F: GGCAGACCAGCATGACAGATT<br>R: GCGGATTAGGGCTTCCTCT
P53	F: TAACAGTTCCTGCATGGGCGGC<br>R: AGGACAGGCACAAACACGCACC
SIN3A	F: ACAGAAGAGGAGAATTCGGATG<br>R: CGCTCCACGTAGTCTGACC
TET1	F: TTCGTCACTGCCAACCTTAG<br>R: ATGCCTCTTTCACTGGGTG
TET2	F: CCCTTCTCCGATGCTTTCTG<br>R: TGGGTTATGCTTGAGGTGTTC
TET3	F: TCCAGCAACTCCTAGAACTGAG<br>R: AGGCCGCTTGAATACTGACTG
TGFBR1	F: CACAGAGTGGGAACAAAAAGGT<br>R: CCAATGGAACATCGTCGAGCA
WT1	F: GGCATCTGAGACCAGTGAGAA<br>R: GAGAGTCAGACTTGAAAGCAGT

Supplementary Figure S1. Gene expression analysis in treatment responsive and refractory AML patients. Cell samples were collected at diagnosis stage and after treatment. Relative gene expression analysis was performed using RT-qPCR ΔΔCt method; GAPDH was used as a "housekeeping" gene. Mean ± standard deviation is presented, grey data points indicate outliers. Mann-Whitney U test was used to determine the significance of difference between groups of different patients' samples, and significance was set at $P \leq 0.05$ (*). Outliers were determined by ROUT ($Q = 5\%$).

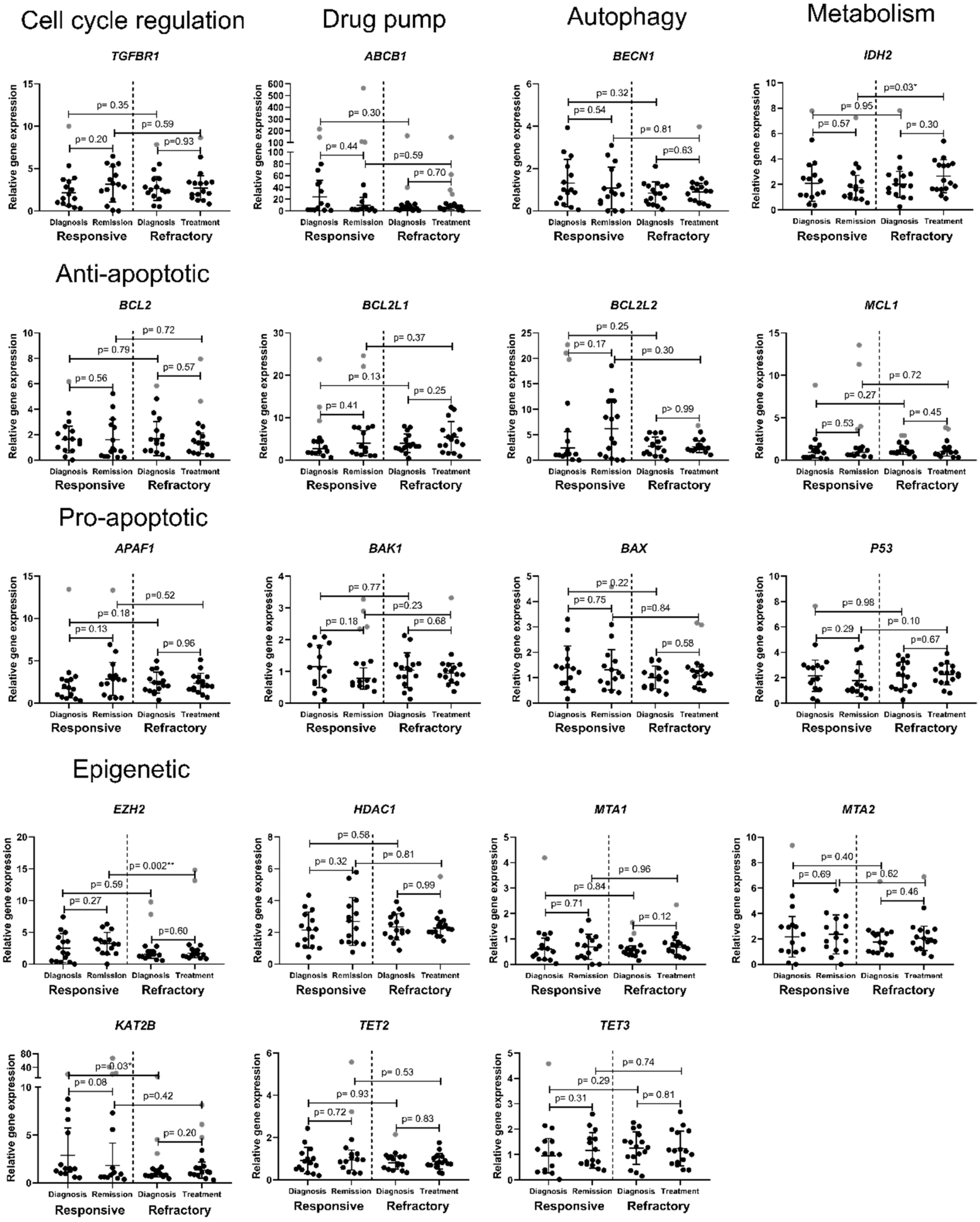

Cell cycle regulation
Drug pump
Autophagy
Metabolism
TGFBR1
ABCB1
BECN1
IDH2
Anti-apoptotic
BCL2
BCL2L1
BCL2L2
MCL1
Pro-apoptotic
APAF1
BAK1
BAX
P53
Epigenetic
EZH2
HDAC1
MTA1
MTA2
KAT2B
TET2
TET3
Relative gene expression
Diagnosis
Remission
Treatment
Responsive
Refractory
p= 0.35
p= 0.59
p= 0.20
p=0.93
p= 0.30
p= 0.44
p=0.59
p= 0.70
p= 0.32
p= 0.54
p= 0.81
p=0.63
p=0.03*
p= 0.95
p= 0.57
p= 0.30
p= 0.72
p= 0.79
p= 0.56
p= 0.57
p= 0.37
p= 0.13
p= 0.41
p= 0.25
p= 0.25
p= 0.17
p= 0.30
p> 0.99
p= 0.72
p= 0.27
p= 0.53
p= 0.45
p=0.52
p= 0.18
p= 0.13
p= 0.96
p= 0.77
p=0.23
p= 0.18
p= 0.68
p= 0.22
p= 0.75
p=0.84
p= 0.58
p= 0.98
p= 0.10
p= 0.29
p=0.67
p= 0.002**
p= 0.59
p= 0.27
p=0.60
p= 0.58
p= 0.32
p= 0.81
p= 0.99
p= 0.96
p= 0.84
p= 0.71
p= 0.12
p= 0.40
p= 0.69
p= 0.62
p= 0.46
p=0.03*
p= 0.08
p=0.42
p= 0.20
p= 0.53
p= 0.93
p= 0.72
p= 0.83
p= 0.74
p= 0.29
p= 0.31
p= 0.81
